# Early laparoscopic cholecystectomy is more cost-effective than delayed laparoscopic cholecystectomy in the treatment of acute cholecystitis

**DOI:** 10.2147/CEOR.S149924

**Published:** 2018-02-19

**Authors:** Doa’a Kerwat, Alexander Zargaran, Reshma Bharamgoudar, Nadia Arif, Grace Bello, Bharat Sharma, Rajab Kerwat

**Affiliations:** 1 Department of Medicine, Barts and The London; 2 Department of Medicine, St George’s University of London, London; 3 Department of Medicine, Birmingham University, Birmingham; 4 Department of Medicine, Brighton and Sussex Medical School, Brighton; 5 Department of Medicine, Imperial College London; 6 Department of Medicine, Queen Elizabeth Hospital, Lewisham and Greenwich NHS Trust, London, UK

**Keywords:** economic evaluation, cost-effectiveness analysis, acute cholecystitis, laparoscopic cholecystectomy, NHS, NICE guidelines

## Abstract

**Background:**

This economic evaluation quantifies the cost-effectiveness of early laparoscopic cholecystectomy (ELC) versus delayed laparoscopic cholecystectomy (DLC) in the management of acute cholecystitis. The two interventions were assessed in terms of outcome measures, including utilities, to derive quality-adjusted life years (QALYs) as a unit of effectiveness. This study hypothesizes that ELC is more cost-effective than DLC.

**Materials and methods:**

In this economic evaluation, existing literature was compiled and analyzed to estimate the incremental cost-effectiveness of ELC versus DLC. Six randomized controlled trials were used to schematically represent the probabilities of each decision tree branch. To calculate health outcomes, quality of life scores were sourced from three articles and multiplied by the expected length of life postintervention to give QALYs. From an National Health Service (NHS) perspective, one QALY may be sacrificed if the incremental cost-effectiveness ratio is above £20,000–£30,0000 in cost savings.

**Results:**

This economic evaluation calculated the average net present values of ELC to be £3920 and DLC to be £4565, demonstrating that ELC is the less-expensive intervention, with potential cost savings of £645 per operation. When scaling these savings up to a population approximately comparable to the size of the UK, full-scale implementation of ELC rather than DLC will potentially save the NHS £30,000,000 per annum.

**Conclusion:**

ELCs are cost-effective from the perspective of the NHS. As such, policy should review existing guidelines and consider the merits of ELC versus DLC, improving resource allocation. The findings of this article advocate that ELC should become a standard practice.

## Introduction

Cholecystectomy is the recommended treatment for acute cholecystitis for over a century and forms a substantial portion of a typical general surgeons’ workload in developed countries.^1^ With the advent of laparoscopic surgery, laparoscopic cholecystectomy has become the gold standard for the treatment of gallstones.^2^ Approximately 57,000 cholecystectomies are performed each year, one-third of which are for the treatment of acute cholecystitis,^3^ and according to Department of Health statistics, 26% of patients undergo early laparoscopic cholecystectomy (ELC) and 74% of patients undergo delayed laparoscopic cholecystectomy (DLC).^4^

The introduction of laparoscopic cholecystectomies in 1987 challenged existing norms regarding best practice of laparoscopic cholecystectomy.^5^ There was an erroneous fear that operating immediately on inflammatory tissue may lead to an increased risk of complications, thereby rendering ELC a contraindication to acute cholecystitis.^6^ However, more recent evidence suggests that ELC does not, in fact, carry a higher risk of morbidity or mortality.^5^

Currently, the National Institute for Health and Care Excellence (NICE) recommends that patients with acute symptoms of cholecystitis should undergo ELC within 1 week of onset, whereas patients deemed medically fit should undergo DLC at a minimum of 6 weeks after they present with symptoms.^7^ However, these guidelines are ambiguous as they do not define the term “medically fit patients”, leaving the decision to the discretion of the clinician. This contrasts with the guidelines in the USA, where ELC is increasingly advocated compared to DLC.^8^

There is a wealth of literature comparing ELC with DLC, some of which suggests that there is a negligible difference in the health outcomes of these two interventions. A Cochrane Review comparing the potential benefits and complications found that both interventions have a similar frequency of postoperative complications with equal mortality rates.^9^ In contrast, recent research and NICE guidelines suggest that ELC has an advantage over DLC and is associated with a reduction in total length of hospital stay, unplanned readmissions, and convalescence.^10^,^11^

With increasing pressure on all aspects of health service delivery, decisions regarding the effective allocation of scarce resources within the NHS are necessary.^12^ This economic evaluation compares the cost-effectiveness of ELC compared to DLC to estimate the total cost and total benefit to the patient. A commonly used method to assess benefit for the patient is the quality-adjusted life year (QALY) that combines both the length and quality of life (QoL). Incremental cost-effectiveness ratio (ICER) was used to determine the most cost-effective intervention.

The hypothesis of this study is to evaluate whether ELC is most cost-effective compared to DLC in the treatment of acute cholecystitis.

## Materials and methods

### Literature review

To obtain accurate data for our cost-effectiveness analysis, a literature review was conducted using three databases: PubMed, EMBASE, and the Cochrane Library. The search string yielded 266 articles in total. Strict inclusion and exclusion criteria were applied to refine the search. Individual articles were further evaluated and excluded depending on their relevance to the specific parameters measured.

### Decision tree

A decision analytic model was formulated to evaluate the cost-effectiveness of two alternatives: ELC versus DLC, for patients suffering from acute cholecystitis. The Markov Model, constructed using TreeAge Pro, provides a schematic of potential treatment choices and associated consequences faced by patients undergoing either treatment or comparator, where the endpoints are clinical outcomes. Probabilities, expected costs, and QoL utilities were sourced from literature and are illustrated on the decision tree. Table 1 focuses on the complications that arise as a consequence of the surgery. From the figures sourced in the literature, expected costs and expected QALYs at each chance node were calculated, working methodically from right to left, while also affording consideration to the probability of each complication arising. Table 1 illustrates the resulting probabilities calculated from the literature.^13^–^17^ The decision nodes were subsequently used to calculate the ICER.

**Figure 1:**
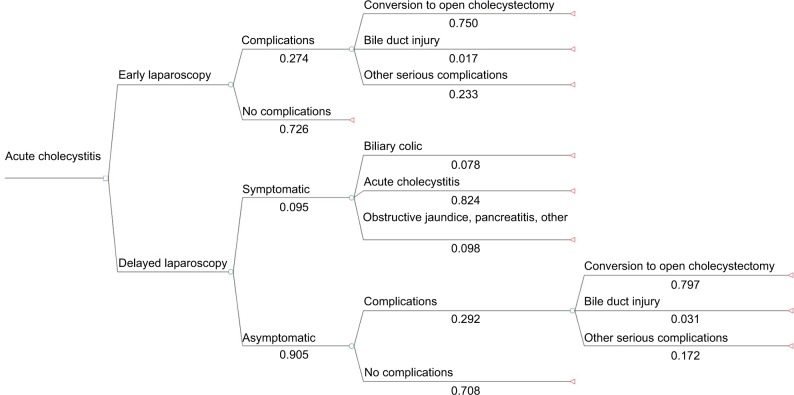
Decision tree - A schematic representation of decisions and the associated consequences of patients undergoing either an ELC or a DLC. **Abbreviations:** DLC, delayed laparoscopic cholecystectomy; ELC, early laparoscopic cholecystectomy.

Clinically significant outcomes are modeled in the decision tree in Figure 1, with chance nodes leading to complications or no complications. For DLC, outcomes are classified as symptomatic and asymptomatic. Complications may result in additional clinical procedures and associated costs, necessitating a comprehensive evaluation of the major clinical permutations arising from ELC and DLC. In this economic analysis, clinical outcomes, costs, and probabilities are considered over the course of a year from initial presentation, which corresponds to the period of time over which a patient is most likely to experience side effects.^18^

The initial decision nodes represent possible alternatives of ELC or DLC for patients with acute cholecystitis. In patients undergoing ELC, the secondary branches reflect the presence of complications such as “conversion to an open cholecystectomy” and “bile duct injury”. Conversely, secondary branches represent symptomatic or asymptomatic patients in patients undergoing DLC. Symptomatic patients are readmitted with exacerbated symptoms, whereas asymptomatic patients exhibit no discernable symptoms. From literature, the most common complications were identified and used to classify outcomes. Symptomatic patients were subdivided into three categories of complications: “biliary colic”, “acute cholecystitis”, and “obstructive jaundice, pancreatitis, other”. Asymptomatic complications were conversion to open cholecystectomy, bile duct injury, and other serious complications.

### Probabilities

Data from the Cochrane Library were utilized, drawing from six separate randomized controlled trials. Of the available trials, one study was excluded owing to lack of clarification by the authors on postrandomization dropouts and incomplete outcome data. The remaining results were collated into a larger sample to provide a high-powered study design, decreasing the risk of Type II error. The final probabilities are shown in Table 1.

### Utilities

QALYs are used to quantify gains in life expectancy and health-related QoL. They take values between 0 and 1, where 1 signifies a year of life that is lived in perfect health, while 0 is death. Length of life (LoL) was found not to differ substantially; therefore, a time horizon of 2 years was multiplied by the reported QoL data found in literature.^19^–^21^ QALYs were not discounted for future gains in health, as health is considered to be neither exchangeable nor investable; thus, 1 year of life is not considered to be of greater value if lived now rather than in the future. Table 2 illustrates the QoL data used in this economic evaluation.

### Costs

Costs in pounds were used as a proxy for resource consumption. Owing to the intrinsic uncertainty and variation in empirical costs faced for each procedure, averages of NHS reference costs for 2014–2015^22^ were used, providing a uniform and up-to-date appreciation of the costs to the NHS. The Complication and Comorbidity (CC) scores were used as a barometer for the severity of each complication, with a score of 5+ reserved for the most severe (conversion to open). An assumption underpinning this economic evaluation is that the reference cost accounts for all relevant associated costs. The additional hospital visit and average of 19 extra minutes of surgery required for “conversion to open” for those under the ELC pathway were accounted for by adding £132 and £270, respectively, with an assumed rate of £850/hour for surgery.^19^ Costs to the hospital are reflective of costs to the NHS, and the final costs used can be found in Table 3. Costs were adjusted for inflation, at a calculated rate of 0.75%.^23^ However, for those costs that are not expected to occur before the end of the 2-year time horizon, discounting was deemed unnecessary.

## Results

The ICER calculated in this analysis for ELC versus DLC was £52,051/QALY, exceeding the willingness-to-accept threshold of £30,000/QALY sacrificed. ELC was calculated to be £645, less expensive than DLC, but was also less effective by 0.012 expected QALYs. Other parameters calculated included a monetary net benefit (MNB) of £273, demonstrating that ELC is more cost-effective when effect is considered only in monetary units, as well as a health net benefit (HNB) of 0.00911, demonstrating that ELC is more cost-effective when only considering health units.

### Sensitivity analysis

Due to small sample sizes used in randomized controlled trials (RCTs), probabilities and costs may be the result of chance, and thus, our ICERs may not reflect the true costs and benefits of ELC versus DLC; therefore, a sensitivity analysis was performed. Four one-way sensitivity analyses were performed on the discount rate, costs, probability of conversion to open cholecystectomy, and probability of symptomatic patients presenting for DLC.

The discount rate of 3.5% stated by NICE guidelines^24^ can take a value of anywhere between 0% and 6% in practice, which were used as the upper and lower bounds for the analysis. Costs were adjusted to find upper (£4412) and lower (£3854) bounds, by using a CC score of 5+ and 0–1 for the most expensive branch (conversion to open). Conversion to open was of particular interest for the sensitivity analysis, as it has been cited as one of the major deterrents for physicians opting to operate early on patients with acute cholecystitis.^25^ Therefore, the third analysis considered the uncertainty surrounding probabilities of conversion to open in ELC. The upper (1.00) and lower (0.33) bounds were obtained from the highest and lowest probabilities in each of the five RCTs, originally collated for data. The final analysis was conducted on symptomatic delayed patients, as these emergency delayed operations are a large reason for why some physicians prefer operating early.^26^ Data were obtained from one of the RCTs^17^ for the upper (0.195) bound and from another^4^ for the lower (0.065) bound by excluding patients for whom it was uncertain whether physicians opted for open surgery before attempting laparoscopic surgery.

**Figure 2:**
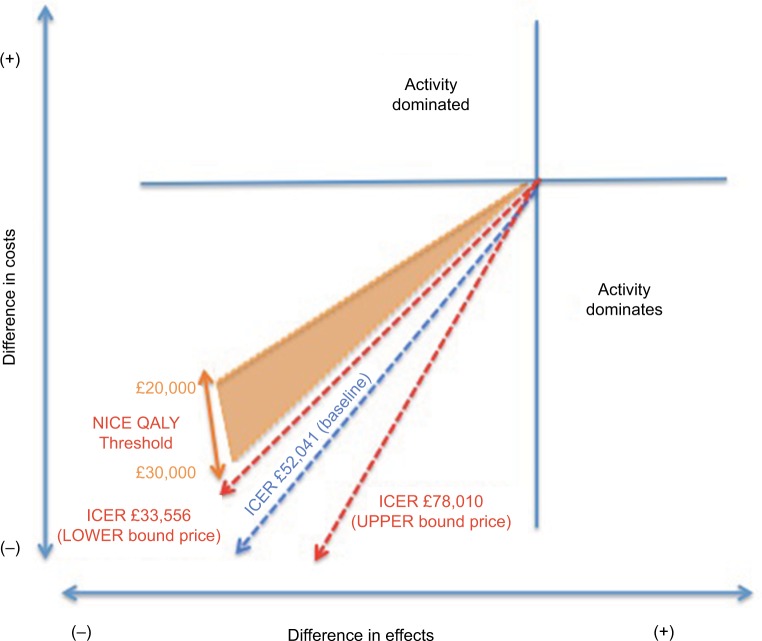
Sensitivity analysis demonstrating the range of ICERs. **Abbreviations:** ICER, Incremental cost-effectiveness ratio; NICE, National Institute for Health and Care Excellence; QALY, quality-adjusted life year.

The sensitivity analysis led to large ranges of ICERs, for example, varied early costs gave values 78,009 > *X* > 33,557. Conversely, sensitivity analysis on the discount rate had a relatively small range of 6,000, demonstrating that the discount rate was less sensitive to change than costs. The decision rule did not change for either analysis, with the ICER still exceeding the minimum willingness-to-accept threshold, rendering ELC cost-effective. However, when probabilities were considered, the sensitivity analysis yielded values far below NICE’s threshold, with ranges of £9,000/QALY for early conversion and £23,000/QALY for symptomatic patients. These two analyses indicate that ELC is not cost-effective and suggest that findings are very sensitive to the probabilities used in the decision tree. These discrepancies between baseline and upper and lower bound values demonstrate the necessity for larger-scale trials to give more reliable data on probabilities. Furthermore, the wide ranges found in the cost sensitivity analysis demonstrate that despite using the most up-to-date costing available through the NHS reference costs, a bottom–up approach is preferable to a more top–down approach, as it would provide a more accurate means of costing and therefore contribute to a more reliable study. Results of the analysis are shown in Figure 2.

## Discussion

This analysis found that each ELC operation could have a potential saving of £645. Assuming that 74% of cholecystectomies are DLC^4^ and using a figure of 57,000 cholecystectomies per year,^3^ this study suggests that the implementation of ELC rather than DLC could save the NHS £27,000,000 per annum. Furthermore, the calculations show an net present value of ELC to be £3,920 and DLC to be £4,565, which also demonstrate that ELC is also the more cost-effective treatment.

The systematic review by Wilson et al^19^ calculated costs using NHS reference costs; their economic evaluation calculated that ELC is approximately £820 less expensive than DLC, concluding that ELC could save the NHS £8.5 million per annum. Moreover, the study by Macafee et al^21^ undertook a CUA of ELC versus DLC using a prospective RCT, which concluded that ELC is less expensive than DLC by £221 from the perspective of both the society and the NHS. They calculated that ELC costs £5,911 compared to £6,132 for DLC. Although our results also draw similar conclusions, using the latest NHS reference costs in our study helps to overcome any discrepancies among figure costs that may have arisen from differences in data sources. More recently, a study published in 2016^27^ presents a cost utility analysis (CUA) that suggests that early cholecystectomy is the optimal intervention compared to delayed cholecystectomy, and although it does not assess laparoscopic surgery specifically, it nevertheless supports our findings as it considers a wider variety of interventions.

This analysis suggests that there is an insignificant difference between the QoL endured by a patient who has undergone early versus late surgery. The results demonstrate a 0.012 difference in QALYs between ELC and DLC, which suggests that ELC may in fact reduce the quality of patient’s lives. This contrasts directly with the literature base. Wilson et al^19^ concluded that ELC is associated with the greatest QALY gains at the least cost compared to DLC and stated that there is a 0.05 QALY gained per patient when undergoing ELC. However, these results lack precision, as they only consider the time horizon of 1 year, whereas this analysis considers a 2-year time period.

Although ELC may be associated with a reduction in QALYs, this study concluded that ELC is cost-effective from the NHS perspective. Savings of £52,000 were calculated, exceeding the minimum of willingness to accept a loss of QALY. As this considers an intervention, which suggests that outcomes are worse, this analysis is concerned with the minimum willingness to accept, rather than the maximum willingness to pay. In contrast, existing studies such as the article by Wilson et al^19^ address the maximum willingness to pay per QALY. While this study offers an alternative position on the cost-effectiveness acceptability graph, it nevertheless draws the same conclusions as existing literature.

As ELC is more cost-effective, we advocate a change in NICE guidelines. A stronger emphasis must be placed on performing laparoscopic cholecystectomies within 1 week of the onset of symptoms. To implement these guidelines, a number of factors must be considered to determine its feasibility. For example, there must be consideration of the potential impact that guidelines may have on both employees and NHS resources, as well as the cultural barriers that may emerge. As this economic evaluation does not propose a new intervention, but instead advocates the discontinuation of one of two existing procedures, and it is unlikely that there will be a significant resistance to change.

Due to budgetary constraints and increasing bed pressures, NHS practice patterns should confer the optimum utilization of resources. As this study is not proposing a new alternative treatment, advocating an early procedure will have little effect on the redeployment of resources. Performing laparoscopic cholecystectomies within a week of presentation may help to alleviate strain on resources because it is associated with significantly fewer readmissions and reduced duration of hospital stay.^15^,^16^ Shorter hospital stays simultaneously reduce the risk of hospital-acquired infections, alleviating strain on the overburdened NHS.^7^ Although society costs are beyond the scope of this economic evaluation, our findings serve to reinforce the conclusions drawn from previously discussed articles demonstrating that ELC benefits both the organization and the society at large.

Despite the efforts made to ensure the reliability of this study, there are a number of limitations. In particular, several assumptions had to be made, as there are few detailed studies in the area of laparoscopic cholecystectomy and the way in which costs were measured. In addition, this study was restricted to a 2-year time frame, as there were limited data available beyond this time; however, literature demonstrates that a majority of complications tend to occur within the first 2 years,^18^ justifying the chosen time frame. Furthermore, complications that may occur beyond this time horizon have not been accounted for. Despite this limitation, other economic evaluations of ELC versus DLC only use a 1-year time horizon, demonstrating that such a limitation is unavoidable in the context of evaluating the cost-effectiveness of laparoscopic cholecystectomies.^19^ Furthermore, a necessary assumption for the decision tree is mutual exclusivity of complications, although in practice, patients may experience more than one complication.

## Conclusion

The latest NICE guidelines include recommendations for both ELC and DLC, with DLC proving to be doctors’ treatment of choice. This analysis has effectively demonstrated that despite a worse health outcome for patients (−0.012 expected QALYs), the cost savings from ELCs exceed NICE’s willingness to accept threshold of £30,000, as demonstrated by an ICER value of £52,014/QALY. HNB and MNB scores support the conclusion that ELC is a more cost-effective treatment than DLC and should, therefore, be made a standard practice in the UK. With NHS resources under intense strain and a growing emphasis on improving efficiency, this evaluation has identified potential savings of nearly £30,000,000 per annum, representing a small step toward a more efficient NHS.

## Figures and Tables

**Table 1:** Probabilities of complications for ELC vs DLC

Branch	Probability
Early laparoscopy	0.259
Conversion to open	0.750
Bile duct injury	0.017
Other serious complications	0.233
Complications	0.274
No complications	0.726
Delayed laparoscopy	0.741
Biliary colic	0.078
Acute cholecystitis	0.824
Obstructive jaundice, pancreatitis, other	0.098
Symptomatic	0.095
Bile duct injury	0.031
Other serious complications	0.172
Complications	0.292
No complications	0.708
Asymptomatic	0.905

**Note:** Data taken from several studies.^13^–^17^

**Abbreviations:** DLC, delayed laparoscopic cholecystectomy; ELC, early laparoscopic cholecystectomy.

**Table 2:** QoL of each decision tree branch

	QoL	Reference
Early laparoscopy		
Conversion to open	0.8	Wilson et al^19^
Bile duct injury	0.8	Wilson et al^19^
Other serious complications	0.71	Wilson et al^19^
No complications	0.91	Bass et al^20^
Delayed laparoscopy		
Biliary colic	0.71	Bass et al^20^
Acute cholecystitis	0.77	Bass et al^20^
Obstructive jaundice, pancreatitis, and other	0.95	Bass et al^20^
Conversion to open cholecystectomy	0.8	Wilson et al^19^
Bile duct injury	0.8	Wilson et al^19^
Other serious complications	0.77	Bass et al^20^
No complications	0.93	Macafee et al^21^

**Abbreviation:** QoL, quality of life.

**Table 3:** Cost data

Index	Branch	Cost (£)
Non-elective, long term	Early laparoscopy – complications, delayed laparoscopy – symptomatic complications	5,300
Non-elective, short term	Early laparoscopy – no complications	3,488
Elective inpatients	Delayed laparoscopy – asymptomatic complications	3,288
Day case	Delayed laparoscopy – no complications	2,057

## References

[1] Dixon E, Fowler DL, Ghitulescu G (2012). Cost-utility analysis of early versus delayed laparoscopic cholecystectomy for acute cholecystitis. Can J Surg.

[2] Ziessman HA (2003). Acute cholecystitis, biliary obstruction, and biliary leakage. Semin Nucl Med.

[3] Royal College of Surgeons of England 2013 Commissioning Guide: Gallstone Disease.

[4] David GG, Al-Sarira AA, Willmott S, Deakin M, Corless DJ, Slavin JP (2008). Management of acute gallbladder disease in England. Br J Surg.

[5] Shikata S, Noguchi Y, Fukui T (2005). Early versus delayed cholecystectomy for acute cholecystitis: a meta-analysis of randomized controlled trials. Surg Today.

[6] Frazee RC, Roberts JW, Symmonds R (1992). What are the contraindications for laparoscopic cholecystectomy?. Am J Surg.

[7] National Institute for Health and Care Excellence (NICE) (2014). Cholelithiasis and Cholecystitis: Diagnosis and Management of Cholelithiasis and Cholecystitis.

[8] Overby DW, Apelgren KN, Richardson W (2010). SAGES guidelines for the clinical application of laparoscopic biliary tract surgery. Surg Endosc.

[9] Gurusamy KS, Samraj K (2006). Early versus delayed laparoscopic cholecystectomy for acute cholecystitis. Cochrane Database Syst Rev.

[10] Gurusamy K, Samraj K, Gluud C, Wilson E, Davidson BR (2010). Meta-analysis of randomized controlled trials on the safety and effectiveness of early versus delayed laparoscopic cholecystectomy for acute cholecystitis. Br J Surg.

[11] Roulin D, Saadi A, Di Mare L, Demartines N, Halkic N (2016). Early versus delayed cholecystectomy for acute cholecystitis, are the 72 hours still the rule? A randomized trial. Ann Surg.

[12] Department of Health (2016). Operational Productivity and Performance in English NHS Acute Hospitals: Unwarranted Variations.

[13] Davila D, Manzanares C, Picho ML, Albors P, Cardenas F, Fuster E (1999). Experience in the treatment (early vs. delayed) of acute cholecystitis via laparoscopy. Cir Esp.

[14] Johansson M, Thune A, Blomqvist A, Nelvin L, Lundell L (2003). Management of acute cholecystitis in the laparoscopic era: results of a prospective, randomized clinical trial. J Gastrointest Surg.

[15] Kolla SB, Aggarwal S, Kumar A (2004). Early versus delayed laparoscopic cholecystectomy for acute cholecystitis: a prospective randomized trial. Surg Endosc.

[16] Lai PB, Kwong KH, Leung KL (1998). Randomized trial of early versus delayed laparoscopic cholecystectomy for acute cholecystitis. Br J Surg.

[17] Lo CM, Liu CL, Fan ST, Lai EC, Wong J (1998). Prospective randomized study of early versus delayed laparoscopic cholecystectomy for acute cholecystitis. Ann Surg.

[18] Machado NO (2011). Biliary complications post laparoscopic cholecystectomy: mechanism, preventive measures, and approach to management: a review. Diagn Ther Endosc.

[19] Wilson E, Gurusamy K, Gluud C, Davidson BR (2010). Cost-utility and value-of-information analysis of early versus delayed laparoscopic cholecystectomy for acute cholecystitis. Br J Surg.

[20] Bass EB, Steinberg EP, Pitt HA (1994). Comparison of the rating scale and the standard gamble in measuring patient preferences for outcomes of gallstone disease. Med Decis Making.

[21] Macafee DA, Humes DJ, Bouliotis G, Beckingham IJ, Whynes DK, Lobo DN (2009). Prospective randomized trial using cost-utility analysis of early versus delayed laparoscopic cholecystectomy for acute gallbladder disease. Br J Surg.

[22] Department of Health [webpage on the Internet] NHS Reference Costs 2014 to 2015.

[23] Statista [webpage on the Internet] Inflation rate: Percentage change on a year earlier of the Consumer Price Index (CPI) in the United Kingdom (UK) from January 2014 to February 2016.

[24] National Institute for Care and Excellence (NICE) (2012). Methods for the Development of NICE Public Health Guidance.

[25] Gul R, Dar RA, Sheikh RA, Salroo NA, Matoo AR, Wani SH (2013). Comparison of early and delayed laparoscopic cholecystectomy for acute cholecystitis: experience from a single center. N Am J Med Sci.

[26] BMJ Best Practice (2018). Acute cholecystitis.

[27] de Mestral C, Hoch JS, Laupacis A (2016). Early cholecystectomy for acute cholecystitis offers the best outcomes at the least cost: a model-based cost-utility analysis. J Am Coll Surg.

